# The myofascial rheostat: hyaluronan molecular weight dynamics and purinergic signalling as a physiological feedback system

**DOI:** 10.3389/fphys.2026.1854585

**Published:** 2026-06-17

**Authors:** Karen B. Kirkness, Fabiana C. da Silva, Robert Schleip, Ronaldo H. Cruvinel-Júnior, Suzanne Scarlata

**Affiliations:** 1 Health Professions Education Unit, Hull York Medical School, York, United Kingdom; 2 Physical Therapy Department, Federal University of Health Sciences of Porto Alegre, Porto Alegre, Brazil; 3 Pain in Motion Research Group, Vrije Universiteit Brussel, Brussel, Belgium; 4 Conservative and Rehabilitative Orthopedics, Technical University of Munich (TUM) School of Medicine and Health, Technical University of Munich, Munich, Germany; 5 Department for Medical Professions, Diploma Hochschule, Bad Sooden-Allendorf, Germany; 6 Experimental Anesthesiology, Ulm University, Ulm, Germany; 7 Department of Physical Therapy, Speech, and Occupational Therapy, School of Medicine, University of São Paulo, São Paulo, Brazil; 8 Department of Chemistry and Biochemistry, Worcester Polytechnic Institute, Worcester, MA, United States

**Keywords:** calcium signaling, CD44/RHAMM, extracellular matrix, fascia, NAD+, HAS2, hyaluronic acid, mechanotransduction

## Abstract

Fascia, the connective tissue network enveloping muscles, organs, and viscera, functions as a hyaluronan (HA)-rich adaptive interface that senses and responds to inflammatory cues. This narrative review synthesizes ECM literature across niches to examine the calcium-hyaluronan (CHA) axis, a feedback loop linking Ca²^+^ signaling to HA synthesis via HAS2 and molecular weight–dependent signaling through CD44 and RHAMM receptors. We propose that the CHA axis is constrained further by ATP/NAD^+^-mediated feedback and functions as a rheostat regulated by four distinct constraint types (1): Mechanical loading and shear regulate HA turnover, hydration, and viscosity via YAP/TAZ and integrin mechanosensing (2). Inflammatory cytokines drive HAS2/HA accumulation during chronic irritation or injury through CD44/RHAMM signaling (3). Metabolic energy availability provides purinergic brakes via AMPK/SIRT1 that suppress synthesis when ATP is scarce (4). HA clearance mechanisms—hyaluronidases, TMEM2/CEMIP, lymphatic drainage, and interstitial fluid flow—which may be as critical as synthesis itself. This is the first review integrating the domains of research across cell types, with clinical relevance in cancer and wound healing as well as exercise physiology and longevity protocols.

## 1 Introduction

### 1.1 HA in fascia: structure, function, and cellular sources

**Figure 1:**
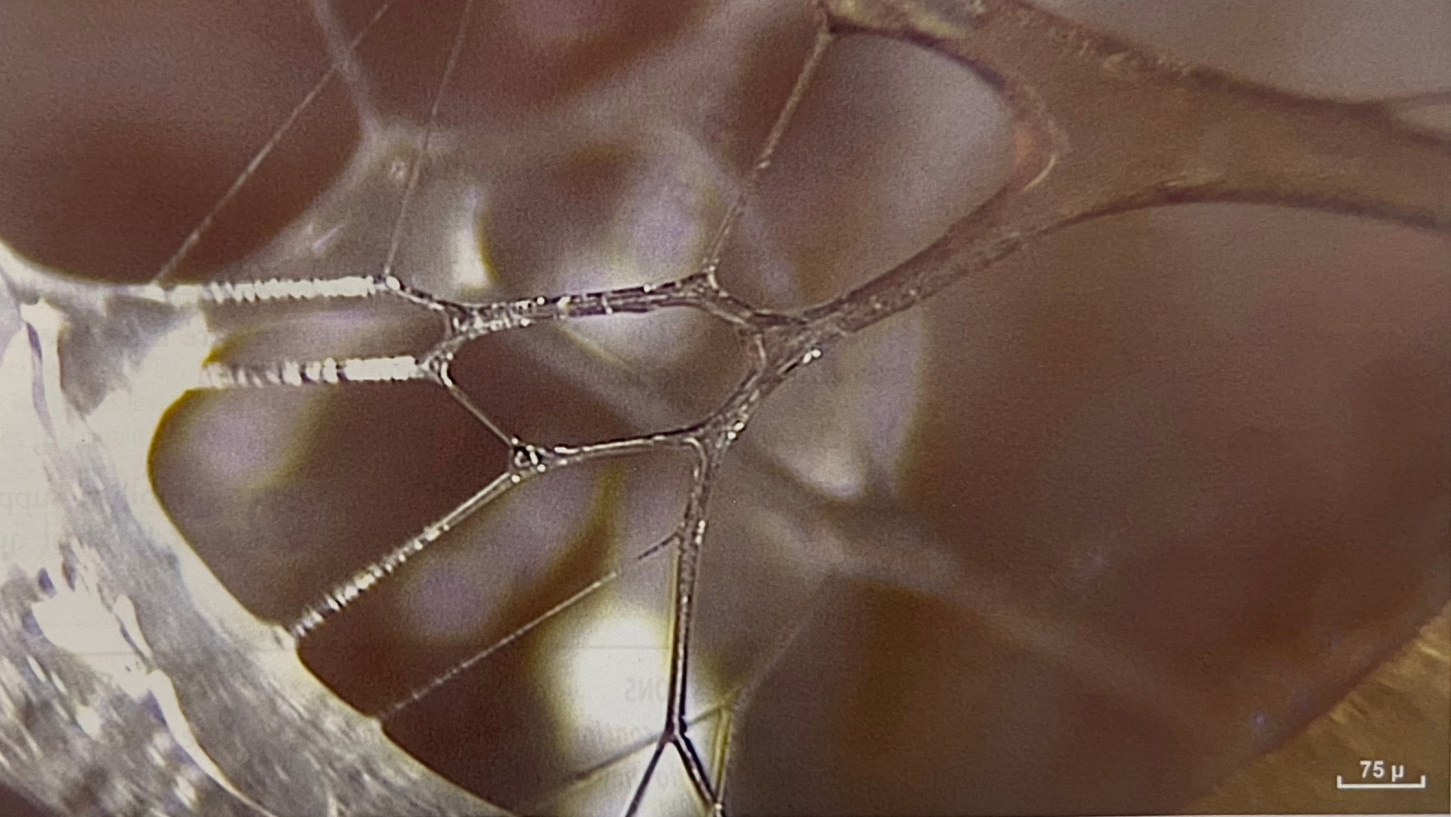
The notion of a structured form is the result of an architectural organization and a continuous, permanent link between all the components. The influence of various physical forces is crucial for maintaining a structured form. Reproduced from Architecture of Human Living Fascia by Jean-Claude Guimberteau (**Figure 2**.25), ^©^ Jessica Kingsley Publishers Limited. Reproduced with permission of the Licensor through PLSclear (Ref. 112545).

Fascia is an integrative tissue consisting of niche cells and their associated extracellular matrix (ECM). The adaptive relationship between cells and ECM enables multisystem regulation, impacting a wide range of physical and even emotional states (Wilke et al., 2018; Zügel et al., 2018; Kodama et al., 2023; Valenti, 2023; Slater et al., 2024; Stecco et al., 2025; Xu et al., 2025). Increasing evidence suggests that ECM adaptation is regulated not only by mechanical loading, but also by metabolic state through pathways involving ATP signaling, AMP-activated protein kinase (AMPK), NAD^+^-dependent sirtuins, and hyaluronan (HA) metabolism (**Figure 1**).

### 1.2 Pathological accumulation of HA

Across many tissues, excess or dysregulated HA (especially fragmented, LMW forms) is linked to inflammation, fibrosis, autoimmunity, and cancer progression (**Table 1**). Metabolic and purinergic HA regulatory mechanisms are well-characterized in other mesenchymal cell types. We propose this work offers tractable application in the myofascial domain where direct experimental evidence remains limited.

**Table 1:** Major tissues where excess hyaluronan associates with disease.

Tissue/organ (cell source)	Pathology/disease context linked to excess HA	Notes on HA role (pro- or anti-disease)
Multiple solid tumors (tumor cells, cancer-associated fibroblasts, myeloid cells)	Breast, prostate, bladder, lung, gastric, glioblastoma, mesothelioma, colorectal, systemic B-cell cancers	High HA matrices and fragments support tumor growth, invasion, angiogenesis, immune suppression, cancer stem traits, poor prognosis (Chanmee et al., 2014; Liang et al., 2016; Heldin et al., 2019; Liu et al., 2019; Nagy et al., 2019; Yang et al., 2019; Donelan et al., 2022; Bao et al., 2023; Donelan et al., 2023; Karalis, 2023; Karousou et al., 2023; Hsu et al., 2025)
Liver (hepatic stellate cells, Kupffer cells)	Liver fibrosis/cirrhosis	HAS2-driven HA in stellate cells drives fibrogenesis, invasion, Notch1 signaling; serum and tissue HA are fibrosis biomarkers (Yang et al., 2019; Kim and Seki, 2023)
Vasculature (arterial smooth muscle cells)	Atherosclerosis, diabetic macroangiopathy	HAS2-mediated HA overexpression in tunica media promotes arterial stiffening and atherosclerosis (Chai et al., 2005; Siiskonen et al., 2015; Yang et al., 2019; Hsu et al., 2025)
Adipose tissue (adipocytes)	Metabolic disease/obesity, T2D context	Adipocyte-specific HA overproduction leads to smaller adipocytes, protection from diet-induced obesity and glucose intolerance (metabolically beneficial) (Siiskonen et al., 2015; Liang et al., 2016; Kobayashi et al., 2020)
Autoimmune thyroid (thyrocytes, thyroid fibroblasts)	Graves’ disease, Hashimoto’s thyroiditis, thyroid-associated ophthalmopathy, dermopathy, hypothyroid myxedema	Thyroid HA accumulation likely contributes to goiter, edema, increased thyroid volume; orbital/skin HA drives ophthalmopathy and dermopathy (Gianoukakis et al., 2007)
CNS (astrocytes, CD4^+^ T cells, microglia)	Multiple sclerosis, EAE, demyelinating lesions	High HA (especially LMW) in lesions promotes astrocyte reactivity, T-cell/microglial activation, impaired repair (Nagy et al., 2019)
Joints/vasculature/lung (various mesenchymal cells)	Atherosclerosis, osteoarthritis, infectious lung disease	HAS1 upregulation and HA accumulation associated with inflammation-driven pathologies (Siiskonen et al., 2015; Hsu et al., 2025)
Gut (intestinal epithelium, stromal and immune cells)	Inflammatory bowel disease (IBD)	Increased HA at inflamed sites modulates immune cell recruitment, cytokine release, coagulation–inflammation crosstalk (Liang et al., 2016; Petrey and de la Motte, 2019)
General connective tissues and organs (broad mesenchymal sources)	Type 1 and 2 diabetes, rheumatoid arthritis, asthma, chronic lung disease, chronic kidney disease, cardiac remodeling, allograft rejection	Elevated HA often correlates with chronic inflammation, fibrosis, worse prognosis across these conditions (Liang et al., 2016; Nagy et al., 2019; Hsu et al., 2025)

## 2 Aims and scope

**Figure 2:**
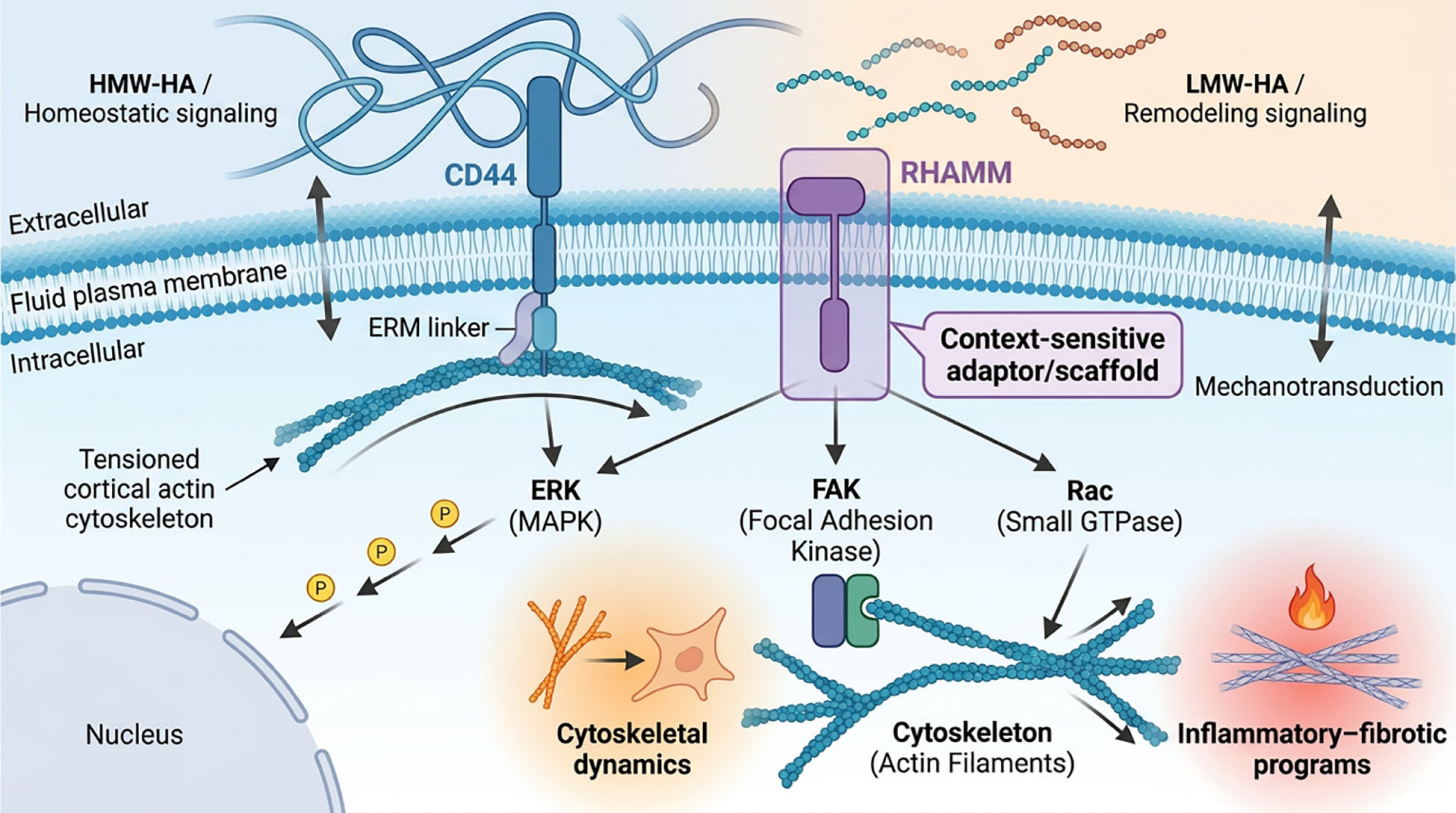
Context-dependent HA signaling through CD44 and RHAMM in mechanobiological ECM regulation. High- and low-molecular-weight hyaluronan (HMW-HA and LMW-HA) engage CD44 and RHAMM through distinct but overlapping signaling architectures that regulate extracellular matrix (ECM) adaptation. HMW-HA preferentially supports CD44-associated homeostatic signaling linked to tissue hydration, cytoskeletal stability, and adaptive mechanotransduction. In contrast, fragmented LMW-HA generated during tissue stress, inflammation, or remodeling preferentially promotes RHAMM-associated signaling and dynamic CD44/RHAMM complexes that activate ERK/MAPK, FAK, Rac, and cytoskeletal remodeling pathways. RHAMM functions primarily as a context-sensitive adaptor/scaffold integrating HA signaling with membrane organization, focal adhesion dynamics, and inflammatory–fibrotic programs.

This narrative review explores molecular mechanisms underlying fascial tissue adaptation with an emphasis on metabolic constraints. Our approach draws from the CHA axis, the tight coupling between Ca²^+^-dependent signaling and HA synthesis with its CD44/RHAMM receptor dynamics (**Figure 2**). Their dynamic reciprocity mirrors other reciprocal regulatory pairs in niche tissues, such as “Go or Grow” epithelial-mesenchymal transition (EMT)/mesenchymal-epithelial transition (MET). The authors previously proposed this as the “Quiet or Riot” reciprocity, a molecular-weight-dependent switching mechanism linking HA signaling to fascial adaptation (Kirkness and Scarlata, 2025). The present review extends this model by integrating the available research on ATP/NAD^+^-mediated feedback into the CHA axis, proposing a physiological rheostat for load-responsive, niche-specific ECM regulation. To our knowledge, this is the first review proposing a metabolically constrained, mechanistic framework for myofascial adaptation.

### 2.1 Conceptual framework

Mechanical stretch induces a nonlinear, threshold-dependent increase in intracellular Ca²^+^ through positive feedback mechanisms that amplify signals once a critical threshold is crossed. Across the retrieved literature, mechanical loads above specific thresholds produce amplified Ca²^+^ signals via positive feedback (Wang et al., 2025; Morrell et al., 2018; Du et al., 2020; Falleroni et al., 2022), while CD44–HA bonds behave as force-sensitive allosteric switches whose affinity and clustering increase under appropriate tension and HA mechanics (Guo et al., (2022); Li et al., 2023; Kim and Kumar, 2014; Suzuki et al., 2015; Jiang et al., 2018; Lintuluoto et al., 2021; Di et al., 2023). At the tissue level, mechanical state transitions mediated by YAP/TAZ and integrin signaling enable tissues to switch responsively among proliferative, homeostatic, and repair states (La Noce et al., 2021; Chu and Quan, 2024).

Collectively, these and many other studies support the view that HA functions as a dynamic signaling regulator within the adaptive, stochastic ECM (Sharkey and Kirkness, 2025). See **Figure 2**. The present review aims to translate these integrated findings from other mesenchymal cell types into the myofascial niche. Burnstock demonstrated that ATP functions not only as an intracellular energy currency but also as an extracellular signaling molecule (Burnstock, 1977; Burnstock, 2002; Burnstock et al., 2013). We propose a conceptual inversion for hyaluronan: HA may function not merely as an extracellular signaling polymer, but as a mechano-metabolic transducer whose synthesis, turnover, and molecular organization convert intracellular energetic state into tissue-scale mechanical and inflammatory behavior.

This integrative framework is informed in part by established work on phospholipase Cβ (PLCβ) signaling, calcium dynamics, and Gαq-mediated membrane mechanobiology. Prior studies by Scarlata and colleagues demonstrated how extracellular receptor activation couples membrane signaling, cytoskeletal remodeling, RNA/protein regulatory systems, and long-term adaptive cellular responses through PLCβ-dependent calcium signaling pathways (Scarlata et al., 2016; Scarlata, 2019; Jackson et al., 2020; Pearce et al., 2020; Rennie et al., 2022). These signaling principles provide an important mechanistic precedent for viewing HA regulation not as an isolated ECM phenomenon, but as part of a broader mechano-metabolic signaling architecture linking energetic state, membrane dynamics, and tissue adaptation.

## 3 HA as fascial matrix foundation

### 3.1 Molecular weight-dependent signaling

HA’s biological effects depend critically on molecular weight: high-molecular-weight HA (HMW-HA) promotes anti-inflammatory, homeostatic responses. Low-molecular-weight HA (LMW-HA) fragments generated during tissue stress trigger pro-inflammatory and reparative signaling (Monslow et al., 2015; Garantziotis and Savani, 2019; Amorim et al., 2021; Kotla et al., 2021; Berdiaki et al., 2023). The distinct receptor affinities for HA fragments of different sizes enable tissues to distinguish signals of damage from those of homeostasis. LMW-HA preferentially binds to RHAMM, acting as a damage signal (Riot mode) that promotes fibrotic and inflammatory responses. In contrast, HMW-HA binds mainly to CD44, signaling tissue integrity and hydration to support repair and anti-inflammatory pathways (Quiet mode).

This molecular weight-dependent signaling allows tissues to distinguish stable conditions requiring maintenance from challenged conditions requiring adaptation. HA properties in fascia (**Table 2**) vary by anatomical region, correlating with mechanical function and required tissue gliding (Fede et al., 2018; Pirri et al., 2022). HA synthesis is primarily mediated by specialized fasciacytes expressing high levels of hyaluronan synthase 2 (HAS2), the enzyme producing HMW-HA (Fede et al., 2018; Pratt, 2021). This enzymatic control of HA production represents a critical regulatory node linking cellular state to ECM adaptation.

**Table 2:** HA’s unique physicochemical properties and their roles in tissue function.

Property/function	Description & mechanism	Tissue/cellular impact	Citations
Water-binding capacity	Binds up to 1000× its weight in water, forming highly hydrated, voluminous networks	Maintains tissue hydration, resists compression, supports ECM	(Monslow et al., 2015; Garantziotis and Savani, 2019; Kotla et al., 2021; Iaconisi et al., 2023)
Viscoelastic behavior	Exhibits both viscous and elastic properties; shear-thinning and shock-absorbing	Facilitates interfascial gliding, joint lubrication, tissue resilience	(Dicker et al., 2014; Monslow et al., 2015; Garantziotis and Savani, 2019; Kotla et al., 2021; Berdiaki et al., 2023)
Cell signaling via CD44/RHAMM	Interacts with CD44, RHAMM, and other receptors to activate signaling pathways (e.g., MAPK, c-Src)	Modulates cell adhesion, migration, proliferation, and response to stimuli	(Dicker et al., 2014; Garantziotis and Savani, 2019; Wolf and Kumar, 2019; Amorim et al., 2021; Kotla et al., 2021; Iaconisi et al., 2023)
Structural/space-filling	Forms extended, entangled networks in ECM	Provides scaffolding, regulates solute diffusion, tissue organization	(Dicker et al., 2014; Monslow et al., 2015; Garantziotis and Savani, 2019; Berdiaki et al., 2023)
Molecular weight-dependent effects	High MW: anti-inflammatory, homeostatic; Low MW: pro-inflammatory, pro-angiogenic	Context-specific modulation of inflammation, repair, and remodeling	(Garantziotis and Savani, 2019; Amorim et al., 2021; Kotla et al., 2021; Berdiaki et al., 2023; Iaconisi et al., 2023)

## 4 Self-limiting purinergic feedback architecture

It is well established that regulated HA turnover as well as molecular weight balance is key to tissue homeostasis. Our previous review aligned the calcium-mediated, molecular weight-constrained receptor loop within the CHA axis (Kirkness and Scarlata, 2025). In the present review, we draw on the available studies in ECM purinergic signaling to evaluate our hypothesis that the CHA axis is also metabolically self-limited.

Purinergic signaling constitutes an extracellular nucleotide-mediated system of cellular communication. Purines are nitrogen-containing aromatic rings that act as building blocks for DNA, RNA, and energy molecules such as ATP. They are found in every cell as they orchestrate tissue responses to stress, damage, and inflammation across diverse physiological contexts.

**Figure 3:**
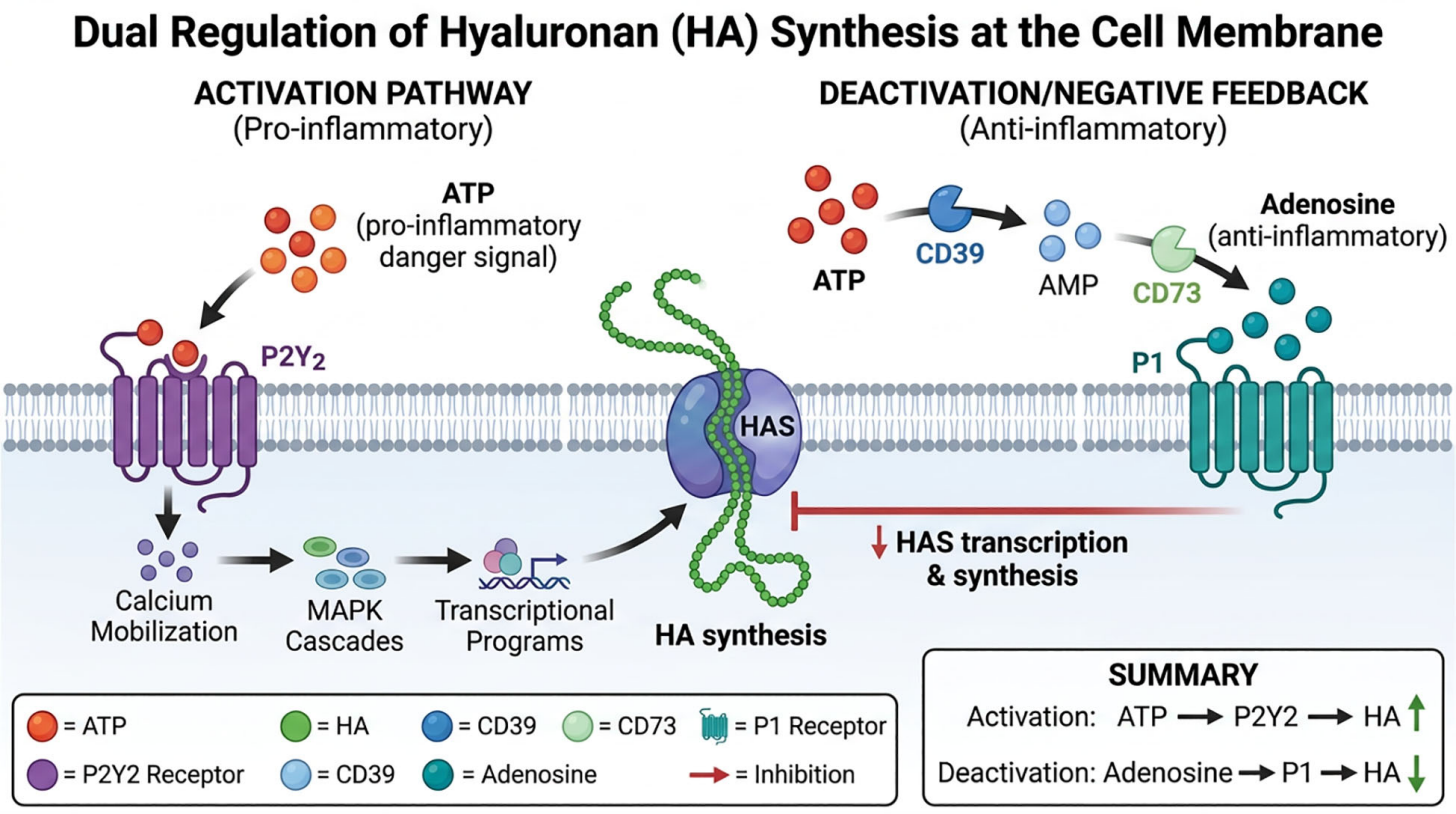
Biphasic purinergic regulation of HA synthesis. #Extracellular ATP released during tissue stress activates P2Y2 receptors, triggering calcium mobilization, MAPK signaling, and transcriptional programs that increase HAS-mediated hyaluronan (HA) synthesis. Sequential ATP degradation by CD39 and CD73 generates adenosine, which activates P1 receptors to suppress HAS2 expression and limit further HA accumulation through delayed antiinflammatory negative feedback. This biphasic ATP→adenosine regulatory circuit is most clearly demonstrated in keratinocytes (Jokela et al., 2017; Rauhala et al., 2018).

At the core of purinergic signaling lies a dynamic ATP–adenosine regulatory axis: extracellular ATP functions as a rapid pro-inflammatory danger signal. See **Figure 3**. ATP binds P2Y receptors (particularly P2Y2) to activate calcium mobilization, MAPK cascades, and transcriptional programs driving cellular synthetic and migratory responses. In turn, its sequential enzymatic degradation by the ectonucleotidases CD39 and CD73 generates adenosine. This anti-inflammatory metabolite terminates ATP signaling and establishes negative feedback through adenosine receptor activation (Eltzschig, 2013; Burnstock and Boeynaems, 2014).

Keratinocytes and the skin barrier represent a logically central site for an HA-regulatory Quiet or Riot switching system such as ATP-adenosine: skin contains the highest HA concentrations of any tissue in the body. See **Figure 3**. Here, epidermal keratinocytes face chronic insult from ultraviolet radiation, mechanical trauma, and recurrent inflammatory stimuli. Such environmental pressures select for tight purinergic feedback control of HA synthesis (Cekic and Linden, 2016; Jokela et al., 2017; Coutinho-Silva and Savio, 2021). Rapid HA extrusion and short half-life in epidermis (2–3 h) and dermis (~1 day) show that skin has multiple clearance avenues to remove HA and its fragments locally and systemically (Kobayashi et al., 2020; Žádníková et al., 2022) after ATP switches on the hose.

## 5 ATP → adenosine: conserved negative feedback

### 5.1 The trigger: ATP release and P2Y2 activation

Under normal conditions, extracellular ATP concentrations remain low (nanomolar range). Mechanical stress, tissue injury, inflammation, or muscle contraction trigger ATP release through vesicular exocytosis, mechanosensitive connexin/pannexin channels, and passive leakage through plasma membrane disruption. These routes collectively generate ATP signals that encode both the intensity of mechanical/injury stress and the transition from acute damage to recovery (**Table 3**).

**Table 3:** Temporal evolution from ATP/P2 to adenosine/P1 dominance.

Phase after ATP release	Dominant species/receptors	Functional bias
Early (seconds–minutes)	High ATP/ADP → P2 receptors	Pro-inflammatory, pro-aggregatory, danger signaling (Covarrubias et al., 2016; Takenaka et al., 2016; Borg et al., 2017; Leite et al., 2023; Shen et al., 2025)
Intermediate	AMP accumulation	Transitional, can dampen some immune functions (Bao and Xie, 2022; Shen et al., 2025)
Later (minutes–hours)	Adenosine via CD73 → P1	Anti-inflammatory, immunosuppressive, tissue-protective (context-dependent) (Antonioli et al., 2013; Takenaka et al., 2016; Losenkova et al., 2022; Gardani et al., 2024; Wang et al., 2025)

This ATP→adenosine conversion is repeatedly described as a time-dependent switch that shortens and limits ATP-driven responses and shapes their magnitude and duration (Trautmann, 2009; Savio et al., 2018; Schädlich et al., 2023; Ikeuchi et al., 2025). A unifying review proposes that route and amount of ATP release scale with force magnitude: mild deformation → vesicular release; stronger strain → hemichannels and resealable tears; catastrophic trauma → bulk ATP spillage (Dsouza et al., 2022).

### 5.2 Adenosine-mediated negative feedback

We may refer to this switch as *temporal damage-response logic*:

- Riot: ATP signals acute injury requiring rapid HA synthesis for barrier repair;
- Quiet: Adenosine acts as a “timer” to prevent excessive accumulation that could promote malignant transformation.

Aortic smooth muscle cells employ their own version of this constraint: AMPK directly phosphorylates HAS2 at Thr-110 in response to low ATP/AMP ratios, suppressing HA synthesis when cellular energy is scarce (Vigetti et al., 2011). This we term as *metabolic energy-sensing logic*. It prevents pathological HA-driven vascular remodeling and atherosclerotic neointima formation, matching the cardiovascular system’s need for sustained metabolic brakes on anabolic ECM synthesis.

**Figure 4:**
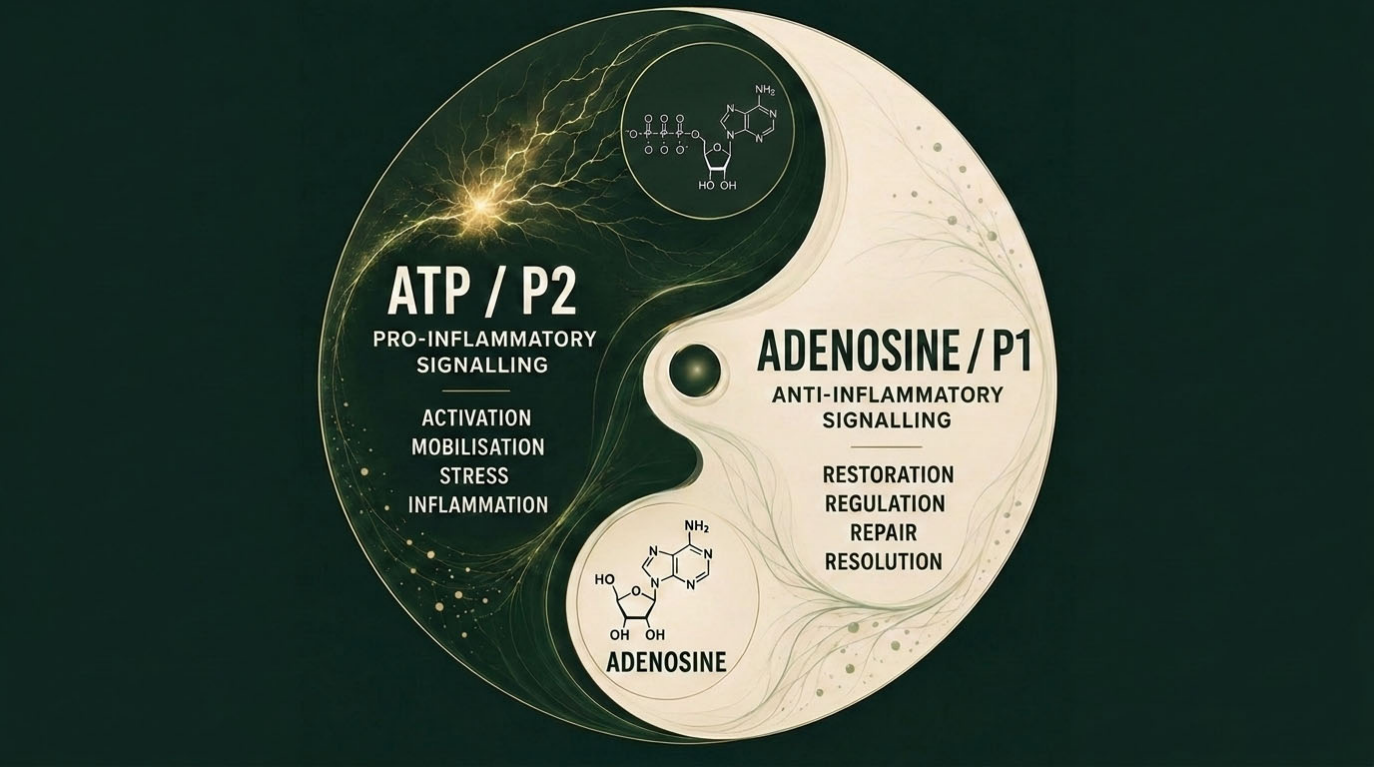
ATP/adenosine yin-yang. Conceptual models emphasize what is repeatedly termed a “Yin–Yang” between pro-inflammatory ATP/P2 and anti-inflammatory adenosine/P1 that “maintain the fine balance” of tissue inflammation in health and are skewed toward excess adenosine in tumors (Faas et al., 2017; Whiteside, 2017; Ecco et al., 2025; Wang et al., 2025).

Both systems prevent pathological HA accumulation, but the strategy varies. Keratinocytes “ask” whether damage has just occurred and is still ongoing, while smooth muscle cells “ask” whether sufficient energy is available to support HA synthesis. These fundamentally different regulatory questions are answered by distinct purinergic mechanisms. Our narrative now turns to the ECM more generally, where such regulatory logic is frequently characterized in the literature as a question of Ying-Yang. See **Figure 4**.

### 5.3 ATP→adenosine: a yin-yang reciprocity

Extensive research across immune and vascular tissues demonstrates that CD39/CD73-generated adenosine reliably shifts purinergic signaling from pro-inflammatory to anti-inflammatory states. The ATP→adenosine pair modulates leukocyte activation, endothelial permeability in tissue remodeling and immune responses (Antonioli et al., 2013; Ferrari et al., 2016). In the vascular system, recent work shows CD39/CD73 limit thrombosis and inflammation and support vasodilation by driving adenosine generation (Kroll et al., 2023; Shen et al., 2025). CD39/CD73 control of the ATP/adenosine ratio functions as a dynamic reciprocity, shifting an ATP-driven inflammatory microenvironment toward an adenosine-dominated state that limits and shapes immune responses rather than simply turning them off (Antonioli et al., 2013; Takenaka et al., 2016; Matyash et al., 2017; Baghbani et al., 2021; Losenkova et al., 2022; Leite et al., 2023; Gardani et al., 2024).

Adenosine via P1 receptors raises cAMP and suppresses T-cell and other effector functions, promoting immune resolution and tissue protection in inflammation, stroke, trauma/hemorrhagic shock, IBD, and cancer models (Trautmann, 2009; Cekic and Linden, 2016; Coutinho-Silva and Savio, 2021; Schneider et al., 2021). This pathway is also repeatedly described as calibrating the duration, magnitude, and chemical nature of purinergic signals delivered to immune cells (Antonioli et al., 2013; Borg et al., 2017; Baghbani et al., 2021). In kidney, CD39 is called a “rheostat” that influences outcomes of acute vs chronic inflammation by modulating ATP vs adenosine over time (Dwyer et al., 2020). Reviews emphasize that, like LMW-HA, extracellular ATP is a general DAMP in immune and vascular beds, with P2 receptors widely expressed and capable of shaping acute vs chronic inflammation (Trautmann, 2009; Di Virgilio and Vuerich, 2015; Cekic and Linden, 2016; Faas et al., 2017; Dosch et al., 2018; Vultaggio-Poma and Di Virgilio, 2022; Di Virgilio et al., 2023). Given that many of these tissues (skin, vessel wall, inflamed stroma) are HA-rich, this provides strong mechanistic plausibility for a generalizable ATP-sensitive HA rheostat. This review will now explore the evidence showing how each ECM niche may have its own tissue-specific rheostat.

## 6 HA homeostasis: from skin to vascular smooth muscle

### 6.1 Damage control: ATP→AMP

Within the provided literature, only keratinocytes show a fully documented ATP→P2Y2→HAS2/HA feed-forward phase followed by AMP/adenosine-mediated suppression of HAS2 and HA as built-in negative feedback (Rauhala et al., 2018). Further, Rauhala et al. appear to be one of the very few studies to experimentally demonstrate *both phases together* in the same keratinocyte system (Rauhala et al., 2018). Other studies have long confirmed the centrality of purinergic signaling in skin physiology and wound healing (Burrell et al., 2005; McEwan et al., 2021). Extracellular ATP was experimentally confirmed as a dominant messenger that forms intercellular Ca^2+^ waves in keratinocytes in 2004 by Koizumi et al. (2004). The importance of purinergic signaling for epidermal homeostasis is corroborated in related research, but studies often focus on single phases or related pathways (e.g., P2X7’s role in inflammation/apoptosis rather than matrix synthesis) (Moehring et al., 2018; Soszyńska et al., 2025).

Other tissues robustly demonstrate (1) mechanical or inflammatory ATP release and P2-driven responses (Dosch et al., 2018; Wei et al., 2019) (2) critical roles of HA–CD44/RHAMM in fibrosis, cancer, and myogenesis (Matou-Nasri et al., 2009; Misra et al., 2015; Leng et al., 2019; Menko et al., 2022), and (3) mechanosensitive HA remodeling in musculoskeletal and fascial contexts (Wei et al., 2019; Fede et al., 2020; Pratt, 2021; Ugwoke et al., 2022; Chan et al., 2024). Our review now turns to the evidence from the smooth muscle niche, particularly the work by Vigetti et al. in cardiac cells (Vigetti et al., 2011). This step helps our narrative contextualize why mapping the purinergic constraints on HA regulation across tissues has been an elusive project.

### 6.2 Metabolic “brakes”: AMPK/SIRT1 and nutrient sensing

#### 6.2.1 Energetic cost and UDP-sugar bottleneck

HA synthesis at the plasma membrane by HAS1–3 consumes large amounts of ATP, UTP, NAD(P)H and acetyl-CoA, making it one of the most energy-demanding anabolic processes in the cell (Moretto et al., 2015; Vigetti et al., 2011; Rauhala et al., 2018; Caon et al., 2020). The cytosolic pools of UDP-GlcUA and UDP-GlcNAc are critical rate-limiting precursors that integrate inputs from glucose, amino acid, lipid and nucleotide metabolism (Moretto et al., 2015; Vigetti et al., 2011; Rauhala et al., 2018; Caon et al., 2021). Changes in UDP-HexNAc also feedback on HAS2 transcription via O-GlcNAcylated transcription factors YY1 and SP1 (Jokela et al., 2017).

#### 6.2.2 AMPK and SIRT1: coordinated metabolic brakes on HAS2

**Figure 5:**
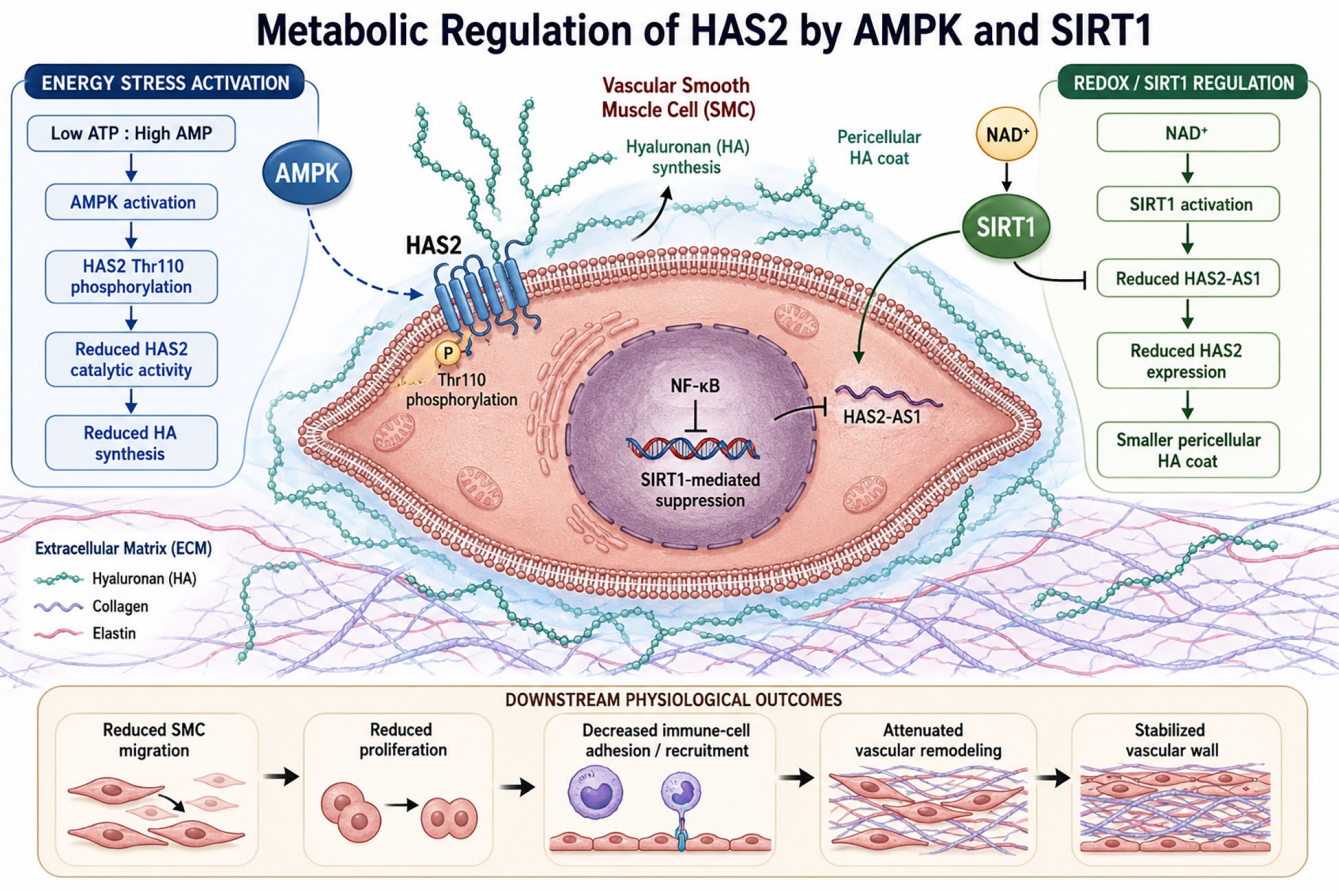
Metabolic regulation of HAS2 and hyaluronan synthesis in vascular smooth muscle cells. AMPK and SIRT1 integrate energetic and redox stress signals to selectively suppress HAS2 activity and HA synthesis in human aortic smooth muscle cells through complementary catalytic and transcriptional mechanisms, reducing pro-atherosclerotic cellular remodeling (Vigetti et al., 2011).

In human aortic smooth muscle cells, AMPK and SIRT1 form an integrated system that functions as a brake-on-brake mechanism. This dual-layer control couples HA output to both cellular energy and redox status. When AMPK is activated—whether by AICAR, metformin, or conditions mimicking low ATP/AMP ratios—it directly phosphorylates HAS2 at Thr110, reducing the enzyme’s catalytic activity. Notably, this inhibition is selective: AMPK suppresses HA synthesis without affecting HAS1/3 or other GAGs (Moretto et al., 2015; Vigetti et al., 2011; Rauhala et al., 2018). The resulting suppression of HA lowers smooth muscle cell proliferation, migration, and immune-cell recruitment, thereby attenuating the pro-atherosclerotic phenotypes that drive vascular disease (Moretto et al., 2015; Vigetti et al., 2011). See **Figure 5**.

Simultaneously, SIRT1 activation—achieved through compounds like SRT1720 or resveratrol—reduces HAS2 expression and pericellular HA coats through a distinct mechanism. Caon et al. demonstrated that SIRT1 prevents NF-κB (p65) from translocating to the nucleus and lowers HAS2-AS1 long noncoding RNA, thereby suppressing HAS2 transcription (Caon et al., 2020). Their study also showed that SIRT1 also blocks TNF-α–induced monocyte adhesion and smooth muscle cell migration by downregulating HA-associated molecules including RHAMM and TSG6 (Caon et al., 2020).

This metabolic-HA receptor interweaving is significant for our argument because it reveals a fundamentally different regulatory architecture than keratinocytes employ. Rather than a temporal toggle (acute damage → ATP burst → adenosine timer), smooth muscle simultaneously dampens HA production while recalibrating the receptors that would amplify its inflammatory signaling. The power of this dual-layer system emerges in its simultaneous action: AMPK phosphorylates HAS2 to lower synthesis; SIRT1 suppresses RHAMM and TSG6 to diminish pro-inflammatory amplification even if HA is present.

AMPK activation raises NAD^+^ availability, which in turn activates SIRT1; SIRT1 reciprocally supports AMPK activity (Cantó et al., 2009; Caon et al., 2020; Caon et al., 2021). This ‘brake-on-brake’ mechanism—metabolic constraint layered atop receptor tuning—prevents pathological vascular remodeling under energetic stress. This reciprocal amplification creates a dual metabolic checkpoint. The aim is to ensure that costly HA synthesis proceeds only when both ATP/AMP ratios AND the redox state (via NAD^+^) are sufficient.

## 7 HA homeostasis in the myofascial niche

Keratinocytes employ rapid ATP→adenosine temporal feedback; smooth muscle employs slow AMPK/SIRT1 metabolic energy-sensing (**Table 4**). Both strategies prevent pathological HA accumulation through fundamentally different physiological logic. For myofascial tissues, we hypothesize a multi-layered constraint system: Are mechanical loading, metabolic capacity, and inflammatory balance aligned to sustain HA turnover and prevent densification?

**Table 4:** Metabolic constraints on HA synthases in smooth muscle cells.

Constraint type	Mechanism affecting HA
UDP-sugar supply	Low UDP-sugars → reduced HA; HAS1 highly sensitive, HAS2 moderately, HAS3 least (Vigetti et al., 2011; Rilla et al., 2013; Vigetti et al., 2014; Kobayashi et al., 2020)
NAD^+^/NADH balance	UGDH reaction alters NAD^+^/NADH; HA synthesis becomes a significant NAD^+^ sink (Vigetti et al., 2014; Caon et al., 2020)
ATP/AMP ratio	Low energy activates AMPK, which phosphorylates HAS2 (Thr110) and inhibits HA synthesis (Vigetti et al., 2011; Vigetti et al., 2014)

### 7.1 The multidisciplinary, priority-optimized HA substrate

Research shows AMPK’s inhibition is selective; it targets HA while sparing other GAGs (Vigetti et al., 2011; Vigetti et al., 2014; Caon et al., 2021). We argue this selectivity is not incidental but reveals a fundamental cellular metabolic triage hierarchy. HA synthesis imposes distinct energetic costs: HAS2 continuously extrudes polymer chains at the plasma membrane in real time. This is an ATP-intensive process requiring sustained substrate flux. In contrast, other GAGs (chondroitin sulfate, keratan sulfate) undergo post-translational modification in the Golgi and covalent attachment to proteoglycan cores—a metabolically distinct, potentially less demanding pathway.

**Figure 6:**
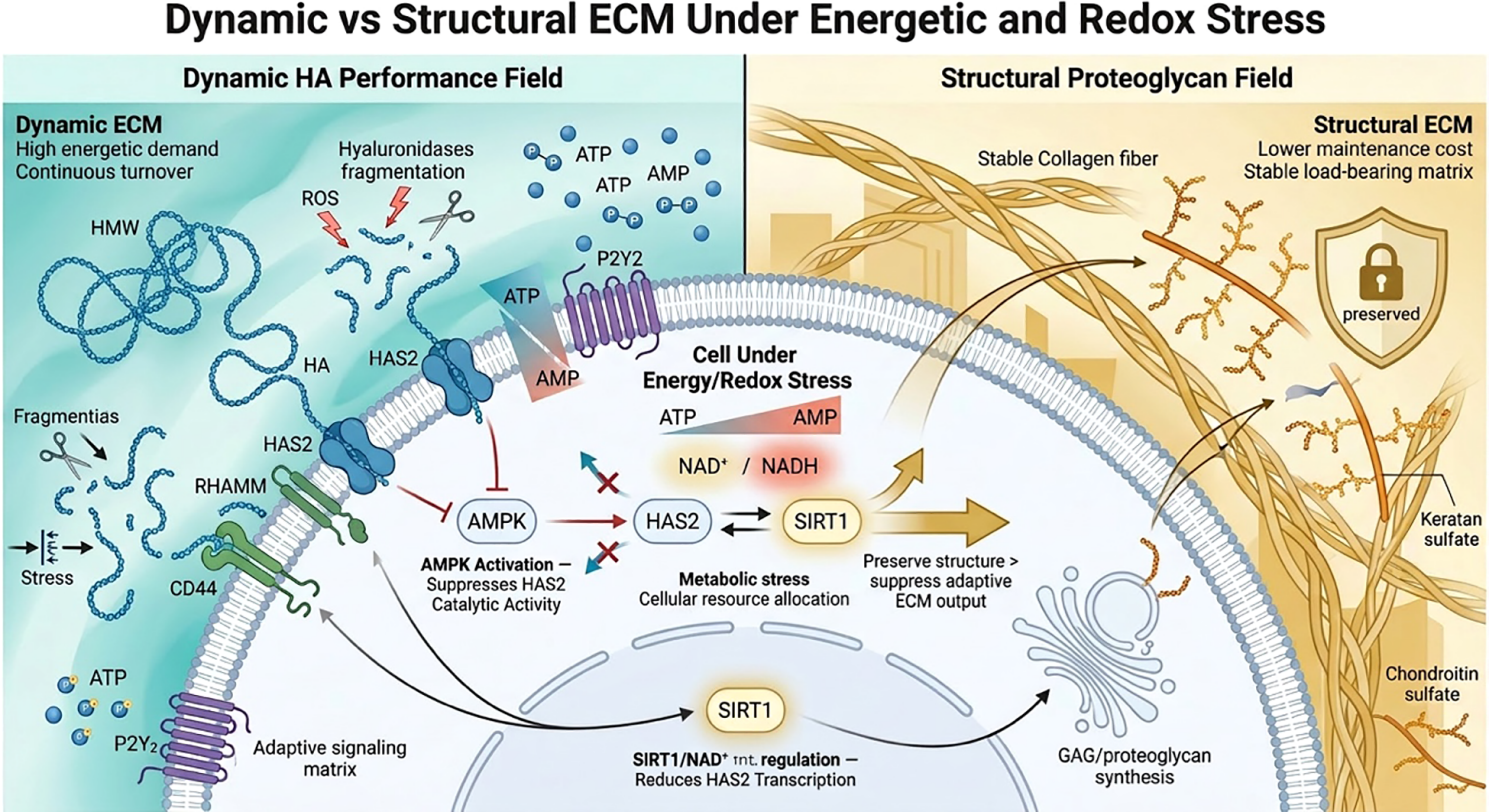
Dynamic and structural extracellular matrix (ECM) systems under energetic/redox stress. HA-rich adaptive signaling matrices and proteoglycan/proteoglycan-linked GAG structural matrices coexist as interwoven but metabolically distinct ECM fields. AMPK and SIRT1 selectively suppress energetically costly HAS2-mediated HA turnover while preserving core structural ECM integrity during metabolic stress.

We posit that, functionally, HA serves as the tissue’s “performance field” enabling viscoelasticity, interfacial lubrication, compliance, and dynamic CD44/RHAMM *and purinergic* signaling (**Figure 6**). Proteoglycan-linked GAGs, by contrast, provide the “structural field,” maintaining osmotic pressure, shape maintenance, and load-bearing integrity essential for cellular geometry. HA’s biological role further demands continuous turnover: hyaluronidase, reactive oxygen species, and mechanical stress fragment HA polymers, necessitating perpetual resynthesis to maintain HMW-HA. Proteoglycan-GAG complexes, once assembled, exhibit greater stability.

For myofascial tissues, this selectivity clarifies a critical point: HA densification under metabolic constraint is not passive accumulation but an active regulatory outcome. When pushed, cells prioritize structural ECM integrity while sacrificing the compliance and signaling field. We argue that the literature demonstrates this as a strategic adaptation that, when prolonged, manifests as pathological stiffness, impaired gliding, and pain.

### 7.2 The consequences of compromise

Contrary to popular belief, too much HA is evidently not better. In the myofascial system, HA behaves like a Goldilocks molecule: too little impairs gliding, but too much—or too viscous/densified—quickly becomes problematic (Stecco et al., 2014; Cowman et al., 2015; Amir et al., 2022; Chan et al., 2024; Raghavan, 2025). Chronic inflammation and immobility risk pathological HA accumulation and densification that impairs gliding, increases viscosity, and causes the stiffness, pain, and microvascular compromise documented in myofascial pain syndrome (Cowman et al., 2015; Stecco et al., 2016), spasticity (Raghavan et al., 2016; Raghavan, 2018), and ischemic myalgia (Bosi et al., 2022; Queme et al., 2022).

The misunderstanding persists that more HA is the answer to stiffness problems. Direct evidence comes from hyaluronidase injection studies shows the opposite: degrading HA reduces stiffness and improves range of motion in spasticity models and post-stroke patients, with effects lasting months without muscle weakness (Raghavan et al., 2016; Stecco, 2016; Mayer, 2018; Raghavan, 2018; Raghavan, 2018; Faivre et al., 2024). This demonstrates that excess HA causes functional impairment and that active HA degradation reverses pathology. Yet no current evidence supports a direct purinergic brake on HAS2 in myofascial tissue. So how does the myofascial niche self-regulate HA turnover?

### 7.3 Purinergic signaling integration across musculoskeletal tissues

Although HA/HAS2 regulation is rarely discussed directly in myofascial research, purinergic signaling is already deeply integrated throughout musculoskeletal mechanobiology (Burnstock et al., 2013). ATP release occurs during contraction, stretch, compression, and fluid shear, with downstream activation of P2X/P2Y receptors, ERK1/2, MAPK, calcium, NF-κB, and inflammatory signaling pathways across skeletal muscle, tendon, cartilage, bone, and connective tissues. Mechanotransductive ATP release via pannexins and hemichannels further establishes local autocrine and paracrine signaling loops that function as energetic regulators of tissue adaptation.

Across connective-tissue and muscle-development models, HA–CD44 (with RHAMM and ERM–actin coupling) controls motility, traction forces, and matrix reorganization (Leng et al., 2019; Cheung et al., 2025). AMPK and SIRT1 are shown to act as energy sensors that selectively dampen HAS2-driven HA synthesis (Rauhala et al., 2018; Caon et al., 2020; Pedrycz-Wieczorska et al., 2025). Together, these pathways are consistent with a graded rheostat linking energetic stress, mechanosensitive signaling, and HA-dependent remodeling, rather than a binary on/off switch, and they provide a mechanistic framework that could extend to myofascial (endomysial/perimysial) matrices.

In this context, the NAD^+^/SIRT1–AMPK system may function in my myofascial niche analogously to a metabolic “resource monitor” (**Table 5**). Here, it may restrain HA synthesis during energetic stress or altered redox conditions. Consistent with this, AMPK activation during low-energy states directly inhibits HAS2 activity and suppresses HA secretion, while SIRT1 activation reduces HAS2 expression, RHAMM signaling, inflammatory activation, and pericellular HA accumulation (Caon et al., 2020; Caon et al., 2021).

**Table 5:** How energy sensors coordinate and limit HA synthesis.

Signal/pathway	Analogy	Main function in HA remodeling
ATP/high energy	Emergency alarm	Enables energy-costly HA synthesis and other anabolic repair when nutrients are abundant (Cantó et al., 2009; Hardie, 2011; Vigetti et al., 2011; Vigetti et al., 2014; Caon et al., 2020; Caon et al., 2021; Chen et al., 2025a)
AMP/low ATP → AMPK	Energy crisis switch	Senses low ATP/AMP ratio and activates AMPK, which phosphorylates HAS2 and inhibits HA secretion to save energy (Cantó et al., 2009; Hardie, 2011; Vigetti et al., 2011; Vigetti et al., 2014; Caon et al., 2020; Caon et al., 2021; Chen et al., 2025a)
Adenosine/AMP	Calming signal/brake	Degradation products of ATP that inhibit HAS2 expression and HA synthesis, helping to shut down an excessive HA response after ATP spikes (Rauhala et al., 2018)
NAD^+^	Fuel meter for SIRT1	Changes in NAD^+^/NADH ratio tune SIRT1 activity, linking redox/energy state to metabolic programs (Fulco and Sartorelli, 2008; Cantó et al., 2009; Zhang and Kraus, 2010; Cantó and Auwerx, 2012; White and Schenk, 2012; Chang and Guarente, 2014; Caon et al., 2020; Yuan et al., 2020; Sánchez-Ramírez et al., 2022; Shen et al., 2024)
SIRT1	Energy budget officer	NAD^+^-dependent sensor that down-regulates HAS2 expression and HA accumulation, partly via NF-κB and HAS2-AS1, and can cooperate with AMPK (Fulco and Sartorelli, 2008; Fulco et al., 2008; Cantó and Auwerx, 2012; Chang and Guarente, 2014; Caon et al., 2020; Yoshihara et al., 2024)
NAD^+^–AMPK–SIRT1 loop	Integrated budget monitor	AMPK activation raises NAD^+^ and activates SIRT1; SIRT1 can, in turn, support AMPK, forming a feedback loop that strengthens catabolism and weakens anabolism to maintain energy homeostasis and restrain costly HA synthesis (Chen et al., 2025b; Fulco and Sartorelli, 2008; Cantó et al., 2009; Cantó and Auwerx, 2012; White and Schenk, 2012; Caon et al., 2020; Nagahisa et al., 2023; Mănescu et al., 2026)

**Figure 7:**
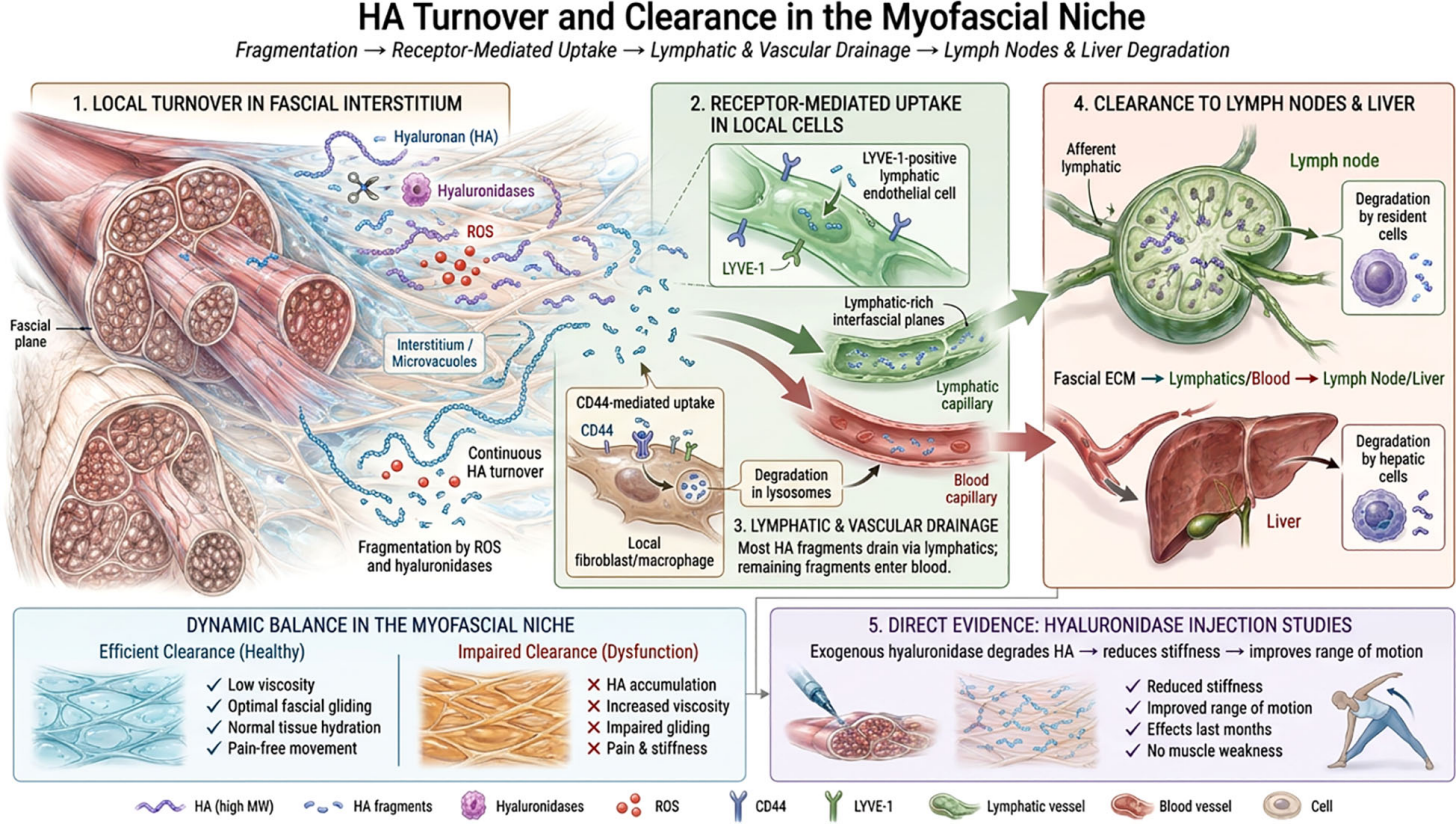
HA clearance in the myofascial ECM.

Excess HA in fascial planes is not simply “stuck.” HA is continuously fragmented by hyaluronidases and ROS, then removed via receptor-mediated endocytosis in local cells, lymph nodes, and liver (**Figure 7**). Efficient lymphatic and vascular drainage are crucial; when clearance is impaired, HA accumulates, viscosity rises, and fascial gliding is compromised. Turnover reviews estimate that only ~10–15% of HA is catabolized locally per day, while the major part is cleared by lymph and blood to lymph nodes and liver for degradation (Laurent and Reed, 1991; Amir et al., 2022).

## 8 The hypothesis: myofascial HA rheostat

### 8.1 Evidence for graded control rather than binary switching

Evidence from fascia, skeletal muscle, and broader musculoskeletal work supports a slower, graded HA-control system closer to the AMPK-SIRT1 of smooth muscle rather than a pure ATP–P2Y_2_ “burst” circuit found in the skin. Skeletal muscle fibers and associated satellite cells express CD44 and are embedded within a HA-rich ECM that undergoes continual remodeling during loading, injury, inflammation, and aging. In smooth muscle cells, HA-CD44 interactions regulate ERK1/2 signaling, migration, and matrix remodeling. Smooth muscle is tightly metabolically gated because it is both contractile and actively matrix-remodeling.

The same purinergic machinery clearly exists across musculoskeletal tissues, and ATP signaling already functions as a mechanosensitive metabolic regulator. However, direct coupling between purinergic signaling and HAS2/HA regulation has not yet been clearly demonstrated in striated muscle, fascia, or tendon ECM. Purinergic–HA crosstalk in striated muscle may be less apparent because the principal ECM-producing cells are distributed among satellite cells, fibro-adipogenic progenitors, immune cells, and connective tissue fibroblasts rather than the contractile myofibers themselves.

### 8.2 Evidence: purinergic-proliferation exists in myofascial niche

Crucially, a study of fascia-derived fibroblasts from the Zusanli acupoint provides the clearest direct evidence that the adenosine-mechanotransduction machinery operates in myofascial tissue (Qu et al., 2019; Tu et al., 2025). The study demonstrated that mechanical stress triggers a measurable metabolic cascade: loading increases energy metabolism, which elevates adenosine levels, which then engages A_3_ adenosine receptors to activate MAPK signaling and stimulate fibroblast proliferation. The observation establishes that the foundational purinergic-metabolic architecture we propose as central to myofascial HA regulation demonstrably exists in the tissue of interest.

Importantly, the Zusanli study did not measure HAS2 expression, HA deposition, CD44, AMPK, or SIRT1 activity in fascia. This apparent gap is resolved by extensive independent evidence: multiple studies directly demonstrate that all these regulatory components are functionally linked in fibroblasts and myocytes (Li et al., 2016; Morris et al., 2025; Power et al., 2025). AMPK and SIRT1 operate in a positive metabolic loop in skeletal muscle and adipocytes: AMPK raises NAD^+^, activating SIRT1, which deacetylates metabolic regulators and can, in turn, support AMPK activity (Cantó et al., 2009; Chau et al., 2010; Sharma et al., 2023).

During embryonic myogenesis, HAS2 is the predominant synthase, and HA, CD44, and RHAMM jointly regulate myoblast and connective tissue cell migration and proliferation, with downstream ERK1/2 signaling (Leng et al., 2019). Vigetti et al. (2011) and Caon et al. (2020) showed that AMPK directly phosphorylates HAS2 at Thr110, suppressing HA synthesis in response to low ATP/AMP ratios in human aortic smooth muscle cells (Vigetti et al., 2011; Caon et al., 2020).

Further, Midgley et al. (2013) demonstrates that CD44 engagement with EGFR and co-localization in lipid rafts is essential for TGF-β-driven myofibroblast differentiation and MAPK/ERK signaling (Midgley et al., 2013). Reiprich et al. (2021) shows that mechanical stress (fluid shear) upregulates HAS2 expression and increases HA secretion in bone marrow-derived mesenchymal stem cells (Reiprich et al., 2020; Reiprich et al., 2021). Across vascular smooth muscle, dermal and synovial fibroblasts, keratinocytes, MSCs, and fascial cells:

- HAS2 expression and HA deposition respond to metabolic sensors AMPK/SIRT1,
- CD44–HA complexes integrate growth-factor and integrin signaling, and
- Mechanical stimuli (shear, stretch, stiffness) modulate HAS2/HA output.

What remains unknown is whether NAD^+^ and adenosine-mediated suppression of HAS2/HA operates in fascial fibroblasts specifically under mechanical loading and inflammatory cues. Given the presence of HAS2^+^ fasciacytes and mechanosensitive YAP and Ang II pathways in fascia (Fede et al., 2018; Caroccia et al., 2025), this hypothesis is grounded in existing evidence (**Table 6**).

**Table 6:** Where key HAS2 regulatory mechanisms are experimentally demonstrated.

Element	Demonstrated in fascia?	Context where shown
HAS2^+^ fasciacytes driving HA	Yes	Human and rat fascia (Fede et al., 2018; Pratt, 2021; Wang et al., 2023)
YAP/Ang II mechanotransduction	Yes	Human deep fascia fibroblasts (Caroccia et al., 2025) and cardiac cells (Niu et al., 2020; Xu et al., 2024) bone marrow stem cells (Reiprich et al., 2021), cancer (Yang et al., 2025) and others
NAD^+^/SIRT1 suppression of HAS2 under inflammation	No (in fascia)	Aortic smooth muscle cells (Caon et al., 2020), human dermal fibroblasts (Sánchez-Ramírez et al., 2022), synoviocytes (Han et al., 2021)
Adenosine-mediated HAS2 inhibition	No (in fascia)	Keratinocytes (Caroccia et al., 2025)

**Figure 8:**
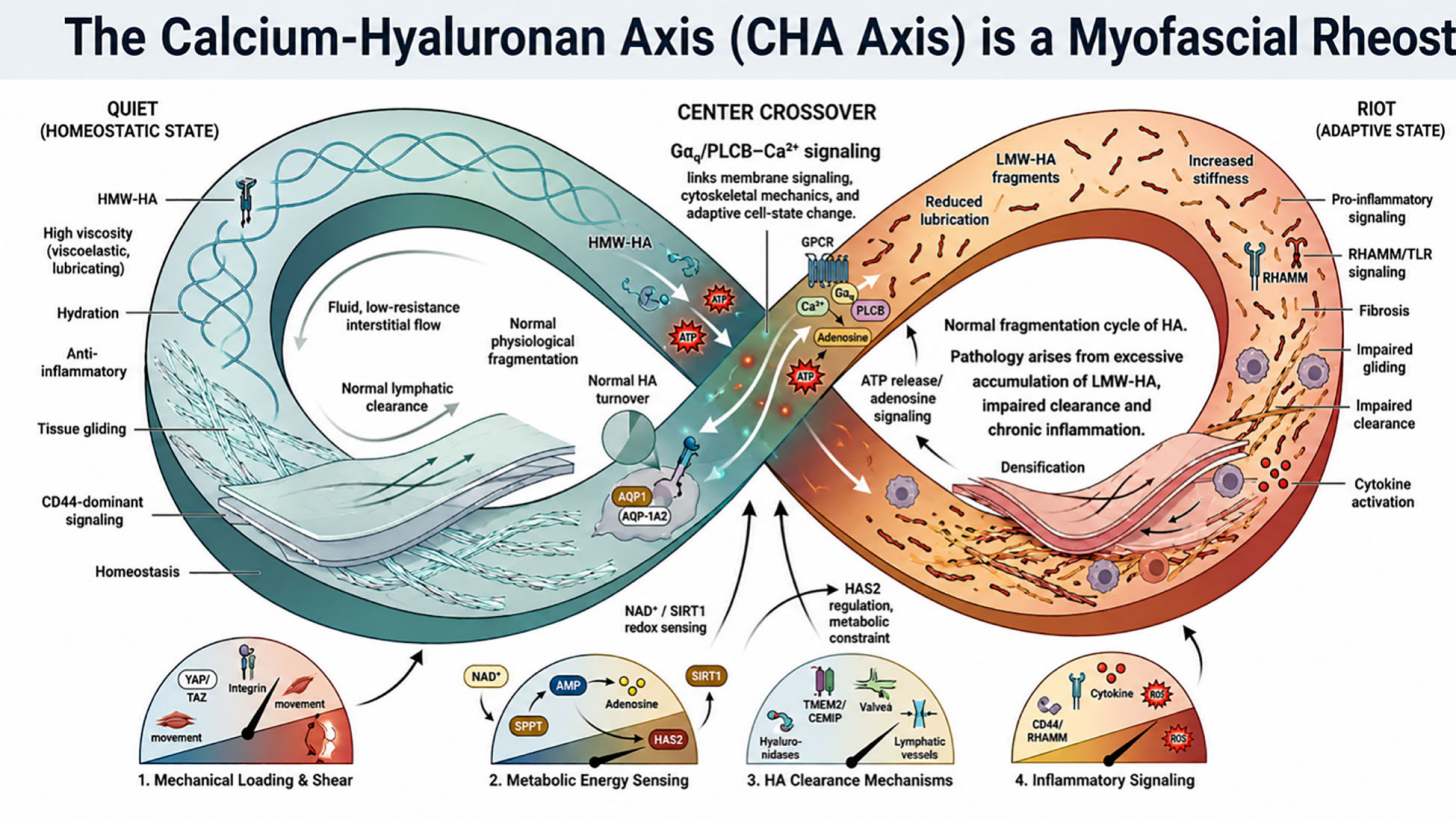
The CHA rheostat. HMW-HA is indeed more viscous than LMW-HA at equal concentration, providing thick, lubricating, viscoelastic behavior. Normal life imposes shear and controlled enzymatic turnover, transiently lowering effective viscosity and aiding movement, while clearance prevents fragment buildup. Clinical HA dysregulation arises when synthesis, fragmentation (often ROS/hyaluronidase-driven), and clearance fall out of balance, leading to accumulation of HA (frequently LMW-rich) that stiffens tissues mechanically and acts as a pro-inflammatory signal. Gq/PLCb is strongly activated by bradykinin and histamine which further drives inflammation.

Extrapolating from the documented tissue-specific regulatory architectures, we propose that myofascia likely employs niche-specific HA constraints within the CHA axis. We propose the term “rheostat” for this homeostatic mechanism, that it oscillates within Quiet/Riot modes according to a matrix of metabolic, mechanical, and inflammatory constraints (**Figure 8**). Our hypothesis rests on established literature; for example, the experimental and review studies from the Karamanos group have demonstrated the importance of HA molecular-weight dynamics and the HAS/HA/CD44 signaling system in context-dependent ECM remodeling, inflammation, fibrosis, and disease progression (Hascall and Karamanos, 2011; Karamanos et al., 2019; Karamanos et al., 2021).

Related work from Nikitovic and collaborators highlighted interactions between HA signaling, oxidative stress, and matrix remodeling (Nikitovic et al., 2013). Garantziotis and Savani emphasized the context-dependent balancing functions of HA across tissue homeostasis and repair (Garantziotis and Savani, 2019). This proportional approach appears better suited to fascia’s physiological challenge: preventing pathological densification under immobility and chronic inflammation, rather than responding to acute barrier breach (as in skin) or variable energy demand (as in vasculature). See **Table 7**.

**Table 7:** Comparative HA regulatory strategies.

Feature	Keratinocytes (ATP/adenosine)	Smooth muscle (AMPK/SIRT1)	Myofascial fascia (mechanical + metabolic)
Trigger	Acute ATP release from mechanical stress, UV damage, or inflammatory insult	Tonic metabolic state; low ATP/AMP ratios during sustained energy demand or ischemia	Reduced mechanical loading (immobility, deload) + sustained low-level inflammation; mismatch between HA synthesis and clearance capacity
Timescale	Rapid (seconds–hours); biphasic ATP→adenosine conversion	Slow (hours–days); tonic sensor activity scales with metabolic state	Slow to intermediate (hours–weeks); scales with immobility duration and inflammatory burden
Question asked	*“Is acute damage happening RIGHT NOW?”* Distinguishes ongoing injury from recovery phase	*“Is sufficient energy available for expensive HA synthesis?”* Couples anabolism to energetic resources	*“Is mechanical stimulus sufficient to prevent HA densification AND is metabolic state adequate for proper turnover?”* Integrates loading history with metabolic capacity
Control lever	Receptor switching (P2Y2→P1 adenosine); ATP activates feed-forward synthesis; adenosine provides negative feedback	Sensor switching (low ATP/AMP→AMPK phosphorylation of HAS2; low NAD^+^→SIRT1 deacetylation of HAS2); direct enzymatic inhibition	Multi-layered (1): mechanical loading via YAP/TAZ-integrin sensing (2); CD39/CD73 upregulation under deload creating adenosine-dominant state (3); AMPK/SIRT1 as secondary metabolic brake (4); hyaluronidase/TMEM2 upregulation for HA clearance
Outcome	Temporal switching (distinguishes injury from recovery); prevents runaway HA accumulation during wound healing	Metabolic constraint (couples HA output to ATP/NAD^+^ availability); prevents pathological synthesis when energy scarce	Densification prevention via coordinated mechanical-inflammatory-metabolic rheostat; fluctuation between Quiet/Riot modes calibrated to loading history and metabolic state
Physiological goal	Prevent malignant transformation from runaway HA accumulation; enable rapid wound healing without excessive ECM deposition	Prevent pathological vascular remodeling and atherosclerotic neointima formation under energetic stress; maintain vascular compliance	Prevent densification-driven mechanical failure; maintain interfascial gliding and compliance layer during immobility and chronic inflammation; preserve tissue resilience across loading cycles

Mechanistically, sustained inflammatory signaling or prolonged absence of mechanical strain could upregulate CD39/CD73 expression, accelerating ATP degradation to adenosine and suppressing HAS2 before HA densification compromises fascial mechanics. This mechanism parallels the logic established in keratinocytes (rapid temporal damage sensing) and smooth muscle (metabolic energy sensing). Each mesenchymal niche evolves an HA constraint matched to its dominant physiological challenge: niche specific temporal damage sensing in skin, metabolic energy sensing in vasculature, and potentially pathological densification prevention in myofascial tissue. The literature demonstrates that ECM remodeling is regulated by the integration of three layers: mechanical loading, inflammatory signaling, and cellular energy availability. SIRT1 and AMPK coordinate these signals by coupling redox state and ATP availability to HAS2 expression, ensuring HA turnover remains matched to metabolic capacity.

### 8.3 Operational definition: from binary switch to proportional dimmer

**Figure 9:**
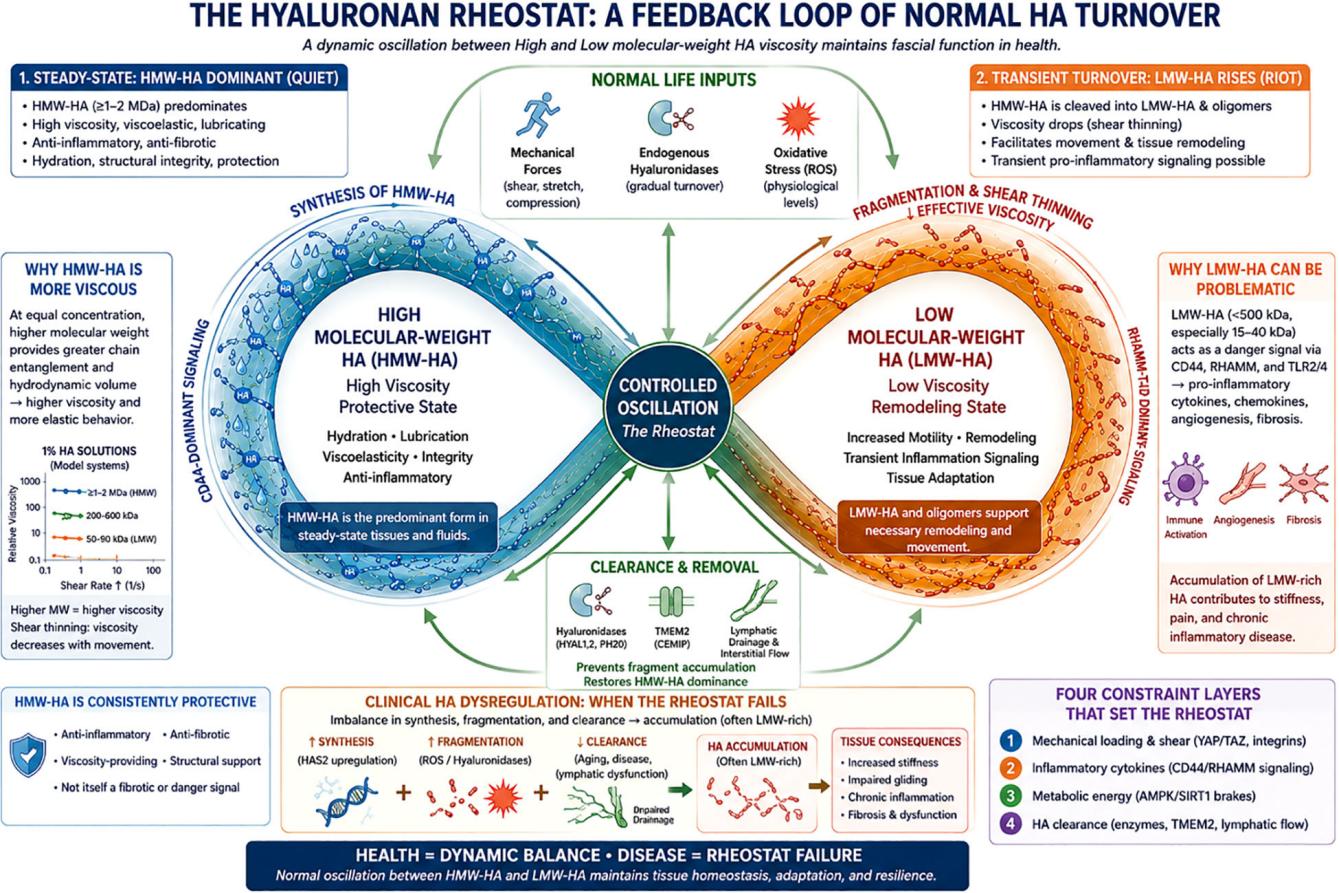
Feedback loop. The rheostat operates continuously, self-adjusting HA synthesis, degradation, and molecular weight distribution in real time according to the tissue’s current physiological state.

Evidence supports the CHA Rheostat as a feedback loop on a dimmer (**Figure 9**). HA turnover in myofascia fluctuates between Quiet/Riot modes not via a single binary switch (as in keratinocytes’ rapid ATP→adenosine timer), but through coordinated, graded constraints. This proportional control creates a response matching tissue state—neither all-on nor all-off—appropriate for myofascia’s unique mechanical and metabolic demands. Similar graded signaling architectures have previously been described in PLCβ-dependent Gαq/Ca²^+^ systems, where membrane signaling, cytoskeletal mechanics, and adaptive stress responses operate through context-sensitive rather than binary regulation (Scarlata, 2019; Jackson et al., 2020; Pearce et al., 2020).

## 9 Limitations and future directions

### 9.1 Limitations

While purinergic signaling is ubiquitous across tissues—and adenosine can modulate immune responses widely—direct suppression of HAS2 by extracellular nucleotides appears restricted to the epidermis based on current evidence. This specificity may reflect the high turnover rate and barrier function requirements unique to skin tissue. The absence of similar findings in fibroblasts or vascular cells suggests either a lack of investigation or fundamental differences in regulatory architecture between cell types. While metabolic sensor, purinergic signaling and HA-CD44/RHAMM dynamics are independently well-characterized in musculoskeletal tissues, no study has directly linked purinergic signaling to HAS2 expression or HA turnover in myofascial contexts. Our “multi-layered rheostat” model is a theoretical framework that has not been experimentally tested in myofascial tissues.

### 9.2 Future directions

Evidence from vascular smooth muscle and keratinocytes shows NAD^+^/SIRT1 and adenosine can suppress HAS2/HA, while fascia studies confirm HAS2^+^ fasciacytes and YAP/Ang II mechanotransduction. What remains untested is whether these metabolic (NAD^+^/SIRT1) and purinergic (adenosine) brakes on HAS2/HA also operate in fascial fibroblasts under mechanical and inflammatory stress, making this a plausible but still hypothetical mechanism.

## 10 Conclusions

This review represents the first integration of purinergic-HA regulatory mechanisms across niche-specific cell types: keratinocytes, vascular smooth muscle, and myofascial tissues. Accordingly, we propose a mechanically-constrained, metabolically-gated rheostat model for myofascial HA regulation. Prior reviews have emphasized HA’s roles in immune signaling, vascular remodeling, or tissue repair in isolation. This framework bridges across niches to highlight a fundamental principle: HA regulatory architecture evolves to match each tissue’s dominant physiological challenge.

## 11 Integration of four regulatory constraints in the myofascial rheostat

This multi-layered rheostat integrates four distinct constraint types:

1. Mechanical loading and shear regulate HA turnover, hydration, and viscosity via YAP/TAZ and integrin mechanosensing. Mechanical deformation activates these pathways, which in turn modulate HAS2 expression and HA clearance mechanisms.
2. Inflammatory cytokines drive HAS2/HA accumulation during chronic irritation or injury through CD44/RHAMM signaling. Sustained inflammatory signals can shift the rheostat toward Riot mode, promoting HA synthesis and remodeling.
3. Metabolic energy availability provides brakes via AMPK/SIRT1 that suppress synthesis when ATP is scarce. This layer ensures that costly HA remodeling does not proceed when cellular resources are depleted.
4. HA clearance mechanisms—hyaluronidases, TMEM2/CEMIP, lymphatic drainage, and interstitial fluid flow—which may be as critical as synthesis itself. Active degradation and removal of HA prevents pathological accumulation independent of synthesis rates.

Additionally, local conditions (temperature, pH, ionic environment, HA concentration, and binding proteins) can shift HA from a low-viscosity lubricant to a densified, viscous matrix that impairs gliding. These four constraints work in concert to maintain fascial compliance and prevent the mechanical failure that characterizes myofascial pain and stiffness.

This framework has direct implications for understanding myofascial pathology and exercise physiology. Pathological HA densification—driven by immobility, chronic low-grade inflammation, or impaired clearance—increases ground substance viscosity, impairs fascial gliding, disrupts microcirculation, and contributes to pain, stiffness, and spasticity. Critically, HA turnover, not merely synthesis, emerges as the therapeutic target. The CHA axis provides a testable mechanistic framework for understanding why exercise recovery depends on metabolic state, why training adaptations fail under energetic insufficiency, and how mechanical loading couples to cellular energy availability through purinergic signaling and NAD^+^-dependent metabolic sensors.
